# Age-dependent effects of homocysteine and dimethylarginines on cardiovascular mortality in claudicant patients with lower extremity arterial disease

**DOI:** 10.1007/s00380-018-1210-9

**Published:** 2018-06-26

**Authors:** Philipp Jud, Franz Hafner, Nicolas Verheyen, Thomas Gary, Andreas Meinitzer, Marianne Brodmann, Gerald Seinost, Gerald Hackl

**Affiliations:** 1 Division of Angiology, Department of Internal Medicine, Medical University Graz, Auenbruggerplatz 15, 8036 Graz, Austria; 2 Division of Cardiology, Department of Internal Medicine, Medical University Graz, Graz, Austria; 3 Institute of Medical and Chemical Laboratory Diagnostics, Medical University Graz, Graz, Austria

**Keywords:** Peripheral arterial disease, Homocysteine, Aging, Intermittent claudication

## Abstract

The association among serum homocysteine (HCY), symmetric dimethylarginine (SDMA), and asymmetric dimethylarginine (ADMA) is of interest in endothelial dysfunction, although the underlying pathology is not fully elucidated. We investigated the relationship of HCY with SDMA and ADMA regarding their long-time outcome and the age dependency of HCY, SDMA, and ADMA values in claudicant patients with lower extremity arterial disease. 120 patients were included in a prospective observational study (observation time 7.96 ± 1.3 years) with cardiovascular mortality as the main outcome parameter. Patients with intermittent claudication prior to their first endovascular procedure were included. HCY, SDMA, and ADMA were measured by high-performance liquid chromatography. Cutoff values for HCY (≤/>15 µmol/l), SDMA (≤/>0.75 µmol/l), and ADMA (≤/>0.8 µmol/l) differed significantly regarding cardiovascular mortality (*p* < 0.001, *p* < 0.001, *p* = 0.017, respectively). Age correlated significantly with HCY (*r* = 0.393; *p* < 0.001), SDMA (*r* = 0.363; *p* < 0.001), and ADMA (*r* = 0.210; *p* = 0.021). HCY and SDMA (*r* = 0.295; *p* = 0.001) as well as SDMA and ADMA (*r* = 0.380; *p* < 0.001) correlated with each other, while HCY and ADMA did not correlate (*r* = 0.139; *p* = 0.130). Patients older than 65 years had higher values of HCY (*p* < 0.001) and SDMA (*p* = 0.01), but not of ADMA (*p* = 0.133). In multivariable linear regression, age was the only significant independent risk factor for cardiovascular death (beta coefficient 0.413; 95% CI 0.007–0.028; *p* = 0.001). Age correlated significantly with HCY, SDMA, and ADMA. However, only age was an independent predictor for cardiovascular death. Older patients have higher values of HCY and SDMA than younger subjects suggesting age-adjusted cutoff values of HCY and SDMA due to strong age dependency.

## Introduction

Lower extremity arterial disease (LEAD) refers to atherosclerotic stenosis or occlusions of the arteries of the lower extremities, which is a disease occurring preferentially in elderly persons. The presence of LEAD might be an indicator of generalized atherosclerosis and therefore poses a significantly increased risk for potentially fatal cardiovascular (CV) events [1]. Known susceptible risk factors are arterial hypertension, hypercholesterolemia, diabetes, and smoking, while the most non-susceptible risk factors are sex and age. Furthermore, there is a known association of these risk factors with endothelial dysfunction due to decreased release of endothelium-derived nitric oxide (NO). Endothelial dysfunction is an independent risk factor of CV morbidity and mortality [2].

The potential role of homocysteine (HCY) in the pathogenesis of CV diseases has been postulated by McCully in 1969. He observed that patients suffering from rare gene defects which led to elevated HCY levels suffered from premature atherosclerosis as early as in their second or third decade of life [3]. At the beginning of the 1990s, the association of HCY and LEAD was more strongly emphasized than the one of HCY and coronary artery disease [4]. By now, the influence of HCY on endothelial function has been thoroughly researched, although the underlying pathology not of the HCY-dependent endothelial dysfunction has not been completely explained yet.

In this context, the association of HCY with symmetric dimethylarginine (SDMA) and asymmetric dimethylarginine (ADMA) is particularly of interest. Contrary to ADMA which directly inhibits the endothelial NO synthase (eNOS), SDMA competes with the intracellular absorption of the NO precursor arginine. This process results in an indirectly decreased NO production via an intracellular deficiency of arginine [5] Both ADMA and SDMA might be directly associated with the occurrence of CV events [6]. HCY is seen as an independent CV biorisk factor as well, although it is unclear whether HCY itself leads to endothelial dysfunction. On one hand, a reduced endothelial-dependent NO liberation due to direct toxic effects on the endothelial cells as well as an inactivation of NO due to increased reactive oxygen species (ROS) production is assumed [7]. On the other hand, there is an assumption that SDMA and ADMA result in an inhibition of eNOS after their activation via HCY and cause an HCY-dependent endothelial dysfunction in this way [8, 9].

The aim of the present study was to investigate the relationship between HCY, SDMA as well as ADMA in claudicant patients with LEAD regarding their long-term prognoses. As HCY metabolism clearly changes with aging, we investigated the age dependency of this relationship in claudicant patients [10–12].

## Materials and methods

### Study design and patient collective

Between March 2002 and November 2004, a total of 120 consecutive patients were included in a prospective observational study with CV-related death as principal outcome parameter. The study included patients who presented at the outpatient clinic of the Division of Angiology in the Medical University of Graz because of intermittent claudication (Rutherford classification stage 2–3) and who had to undergo their first endovascular procedure of the pelvic and/or femoropopliteal arteries due to a significant hemodynamic lesion in the respective arteries despite antiplatelet therapy. All patients were on antiplatelet therapy prior to endovascular intervention with either acetylsalicylic acid 100 mg or clopidogrel 75 mg per day. All patients underwent prior unsupervised exercise therapy, while none of the patients took folate or vitamin B12 supplements due to the influence of both vitamins on HCY metabolism. The patients’ dietary intake was in accordance with an average Middle European diet. Antegrade or retrograde access via the common femoral artery was used in all interventions. Patients suffering from LEAD below the knee objectified by Doppler ultrasound of the arteries or magnetic resonance angiography were not included in the study, as intermittent claudication (Rutherford classification stage 2–3) is not an indication for endovascular recanalization in patients with LEAD below the knee [13]. The occurrences of a fatal stroke or fatal myocardial infarction were defined as CV death. Patients who were suffering from unstable angina pectoris or consequences of a stroke at the time of recruitment were excluded from our study. Other exclusion criteria were uncontrolled arterial hypertension (defined as a blood pressure above 180/120 mmHg at the time of study inclusion after 10 min in resting position), decompensated heart failure, life expectancy of less than a year, wound infections, vegetarians or vegans, and contraindications against anticoagulants and/or antiplatelet agents. All patients gave their written informed consent after being accurately informed about the clinical trial. The study was approved by the Institutional Review Board of the Medical University Graz, Austria (EK 23-038 ex 10/11).

### Data collection

On the day of the endovascular intervention, the patients’ baseline characteristics were determined. Subsequently, a total of four follow-up visits after 1, 3, 6 and 12 months were scheduled. At each study visit, the patients’ concomitant medication and the occurrence of CV events were recorded. The final examination was conducted between October 2010 and May 2011. During the final examination, the occurrence of CV events was recrded. For this purpose, patients were invited to an outpatient examination/survey, in which they answered questions about their LEAD symptoms, medical history, and current medication. Using the same survey, patients who could not participate in the examination were interviewed on the telephone as an alternative means of data collection. If patients were deceased or not reachable by telephone, the primary care physician of the respective patients was contacted and informed about the study. This enabled the collection of the necessary data regarding mortality and the occurrence of CV events (CV death, stroke, myocardial infarction) as well as current medication. Finally, all medical files in all public Styrian hospitals including their emergency rooms and divisions of pathology were reviewed to complete data collection.

### Biochemical analyses

At the baseline visit, fasting blood samples were obtained. The serum was centrifuged and stored at − 70 °C until further analysis of HCY, SDMA, and ADMA was performed in March 2011 by means of high-performance liquid chromatography with a solid phase extraction and precolumn derivatization technique which was first described by Teerlink with only minor modifications [14, 15]. According to previous reports, the investigated biomarkers can be assumed as stable [16]. Within-day coefficients of variation for SDMA were 4.6% (0.60 µmol/L) and 1.9% (1.0 µmol/L), and between-day coefficients of variance were 9.8% (0.60 µmol/L) and 6.1% (1.0 µmol/L). Within-day coefficients of variation for ADMA were 3.1% (0.62 µmol/L) and 1.0% (2.0 µmol/L), and between-day coefficients of variance were 9.0% (0.62 µmol/L) and 2.2% (2.0 µmol/L).

### Statistics

In case of continuous variables, patient characteristics were given as means (± standard deviation). Median and interquartile range were used to express skewed data. Categorical variables were represented by frequency and percentages. The normal distribution was examined via Kolmogorov–Smirnov and Shapiro–Wilk test. The two-sided *t* test was used for the comparison of groups in case of parametrical distribution. For non-parametrical data, a Mann–Whitney *U* test was utilized. Using a *χ*^2^ and Fisher’s exact test, qualitative command variables were compared. Optimal cutoff values for HCY, SDMA, and ADMA as potential predictors of subsequent cardiovascular death were evaluated by receiver operating curve (ROC) analyses. We applied log-rank statistics and assessed survival analysis utilizing Kaplan–Meier curves. The Jonckheere–Terpstra test was used for trend statistics. Correlations between metrical variables were expressed by Pearson´s correlation coefficients. Variables were also assessed as predictors of all-cause mortality in multivariate cox proportional regression analyses. We assumed statistical significance when *P* value was < 0.05. Statistical analyses were executed via SPSS version 20.0.

To assess the age dependency of concentrations of HCY, ADMA, and SDMA, the cohort was split by age of 65 years because the conventional age threshold at which people can be assumed to be “old” is commonly 65 years. Between-group differences were assessed using analysis of variance.

## Results

A total of 120 patients were included in the analysis. Cardiovascular deaths were recorded during a mean follow-up of 7.96 (± 1.3) years. Patients´ baseline characteristics are shown in Table 1.

**Table 1:** Patients’ characteristics

*N*	120
Men, *n* (%)	84 (70.0)
Age (y), mean (± standard derivation)	66.5 (± 10.7)
BMI in kg/m^2^, mean (± standard derivation)	26.7 (± 3.2)
Observation time in years, mean (± standard derivation)	7.96 (± 1.3)
Previous history, *n* (%)	
Myocardial infarction	11 (9.3)
Cerebrovascular disease (stroke, TIA)	8 (6.8)
Diabetes mellitus	38 (31.7)
Arterial hypertension	94 (78.3)
Smoking	77 (73.3)
Current	48 (40.0)
Ex	42 (35.0)
Hypercholesterolemia	60 (50.0)
Kidney function	
eGFR, ml/min^−1^ 1.73 m^−2^, mean (± standard derivation)	68.3 (± 24.6)
	1.2 (1.0–1.3)
Creatinine, mg/dl, median (25th–75th percentile)	
ABI, median (25th–75th percentile)	0.70 (0.57–0.89)
Interventional procedure, *n* (%)	
PTA	98 (81.7)
PTA + stent	22 (18.3)
Drug therapies at discharge, *n* (%)	
Antiplatelet agents	102 (85.0)
Beta-blockers	37 (30.8)
ACE inhibitors or ARBs	71 (59.1)
Statins	55 (45.8)
Hemoglobin in g/dl, mean (± standard derivation)	13.9 (± 1.6)
Platelets in G/l, mean (± standard derivation)	235 (± 59)
hs-CRP in mg/l, median (25th–75th percentile)	4.0 (2.1–7.3)
Homocysteine in µmol/L, median (25th–75th percentile)	14.7 (12.1–17.5)
Lipids in mg/dl, median (25th–75th percentile)	
LDL	120 (93–143)
HDL	47 (40–59)
Triglycerides	150 (105–206)
Lipoprotein (a)	12.5 (9.5–42.0)
HbA1c in %, median (25th–75th percentile)	5.7 (5.4–6.4)

*BMI* body mass index, *TIA* transient ischemic attack, *eGFR* estimated glomerular filtration rate, *ABI* ankle–brachial index, *PTA* percutaneous transluminal angioplasty, *ACE* inhibitors angiotensin-converting enzyme inhibitors, *ARB* angiotensin-receptor blockers, *hs*-*CRP* high-sensitive C-reactive protein, *LDL* low-density lipoproteins, *HDL* high-density lipoproteins, *HbA1c* hemoglobin A1c

**Fig. 1:**
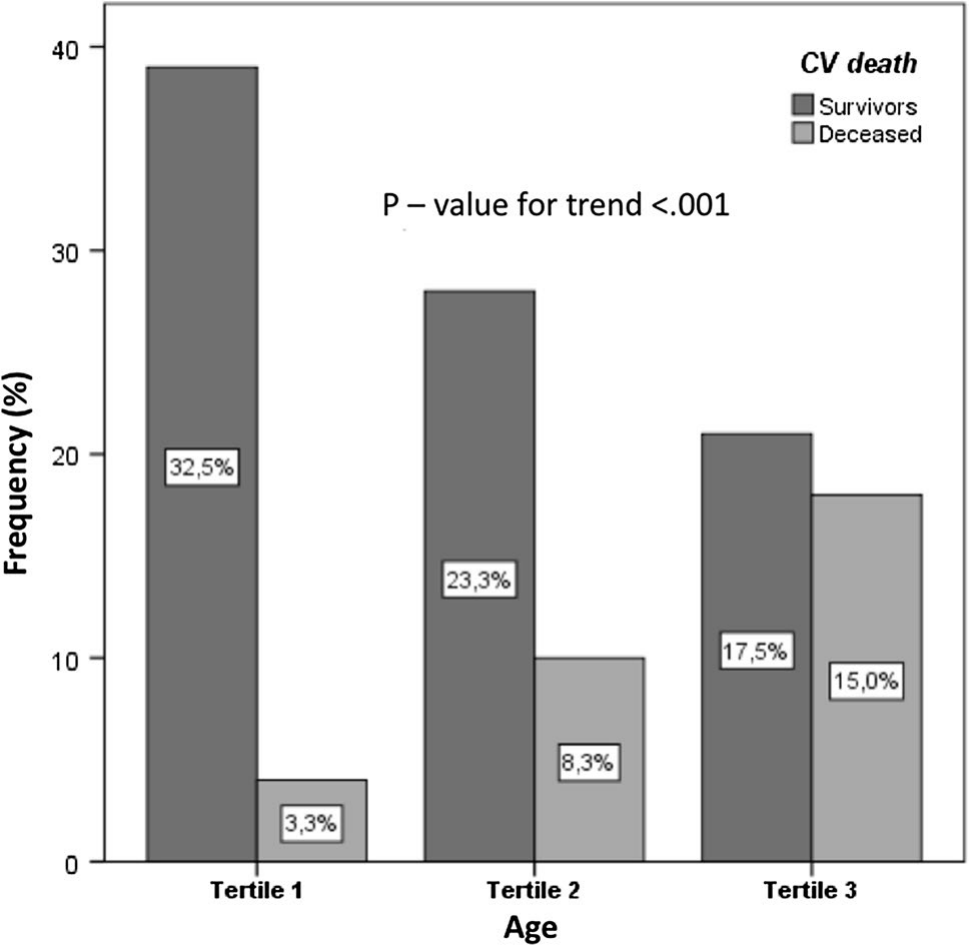
Tertiles of age stratified with cardiovascular death

At the beginning of our analysis, age was divided into tertiles (first tertile: 54.93 ± 6.39 [95% CI 52.96–56.90] years; second tertile: 67.53 ± 2.90 [95% CI 66.57–68.48] years; third tertile: 78.23 ± 3.22 [95% CI 77.19–79.27] years). These tertiles were compared with respect to CV death. As expected, a significant difference between the three groups was observed (Fig. 1). Subsequently, the optimal cutoff value for HCY, SDMA, and ADMA between surviving and deceased patients was determined utilizing ROC curves. The value of HCY was 15 µmol/l, the one of SDMA 0.75 µmol/l, and the one of ADMA 0.8 µmol/l (Table 2).

**Table 2:** ROC analyses evaluating cutoff values for HCY, SDMA, and ADMA

Variable	Area ± SE	Asympt. Sign.	95% CI	Cutoff value (µmol/l)	Sensitivity	Specificity
HCY	0.637 ± 0.059	**0.022**	0.522–0.751	15	0.656	0.602
SDMA	0.670 ± 0.060	**0.005**	0.553–0.787	0.75	0.594	0.682
ADMA	0.632 ± 0.061	**0.028**	0.512–0.752	0.8	0.375	0.898

*p* values < 0.05 are shown in bold

*HCY* homocysteine, *SDMA* symmetric dimethylarginine, *ADMA* asymmetric dimethylarginine

In the next step, the groups were compared with the established cutoff values. Age was significantly different for all three variables (HCY *p* < 0.001, SDMA *p* < 0.001, ADMA = 0.017, respectively) (Table 3).

**Table 3:** Comparison of population with HCY ≤/>15 µmol/l, SDMA ≤/> 0.75 µmol/l and ADMA ≤/> 0.8 µmol/l

Variable			*P* value
	HCY ≤ 15 µmol/l	HCY > 15 µmol/l	
Men, *n* (%)	47 (39.2)	37 (30.8)	0.842
Age (y)	63.2 (± 10.0)	70.5 (± 10.2)	**<** **0.001**
BMI (kg/m^2^)	27.0 (± 3.0)	26.4 (± 3.4)	0.354
eGFR (ml/min^−1^ 1.73 m^−2^)	76.1 (± 23.3)	58.8 (± 22.8)	**<** **0.001**
ABI	0.72 (± 0.26)	0.68 (± 0.41)	0.398
Hemoglobin (g/dl)	14.3 (± 1.4)	13.4 (± 1.8)	**0.002**
Platelets (G/l)	226.9 (± 47.0)	239.9 (± 71.2)	0.253
CRP (mg/l)	4.5 (± 3.1)	4.4 (± 3.2)	0.491
Total cholesterol (mg/dl)	210.7 (± 44.0)	199.6 (± 51.1)	0.203
LDL (mg/dl)	127.1 (± 35.6)	111.5 (± 41.5)	**0.030**
HDL (mg/dl)	48.0 (± 19.5)	45.0 (± 20.0)	0.329
Triglycerides (mg/dl)	166 (± 10)	174 (± 14)	0.738
HbA1c (%)	5.7 (± 0.9)	5.7 (± 1.1)	0.734
	SDMA ≤ 0.75 µmol/l	SDMA > 0.75 µmol/l	
Men, *n* (%)	52 (61.9)	32 (38.1)	0.839
Age (y)	63.5 (± 10.4)	71.2 (± 9.5)	**<** **0.001**
BMI (kg/m2)	26.9 (± 3.2)	26.4 (± 3.1)	0.423
eGFR (ml/min^−1^ 1.73 m^−2^)	75.9 (± 25.2)	56.4 (± 18.1)	**<** **0.001**
ABI	0.69 (± 0.36)	0.79 (± 0.39)	0.544
Hemoglobin (g/dl)	14.1 (± 1.6)	13.5 (± 1.7)	0.061
Platelets (G/l)	239.1 (± 63.9)	223.0 (± 50.3)	0.146
CRP (mg/l)	4.4 (± 3.0)	4.5 (± 3.4)	0.496
Total cholesterol (mg/dl)	208.2 (± 45.5)	201.9 (± 50.6)	0.479
LDL (mg/dl)	125.5 (± 41.1)	111.5 (± 34.4)	0.058
HDL (mg/dl)	49.0 (± 20.0)	44.5 (± 17.8)	0.946
Triglycerides (mg/dl)	170 (± 10)	168 (± 15)	0.780
HbA1c (%)	5.7 (± 1.0)	5.8 (± 0.9)	0.281
	ADMA ≤ 0.8 µmol/l	ADMA > 0.8 µmol/l	
Men, *n* (%)	72 (85.7)	12 (14.3)	0.192
Age (y)	65.4 (± 10.6)	71.5 (± 10.0)	**0.017**
BMI (kg/m2)	26.9 (± 3.1)	26.0 (± 3.3)	0.276
eGFR (ml/min^−1^ 1.73 m^−2^)	70.1 (± 24.1)	59.7 (± 25.6)	0.076
ABI	0.71 (± 0.30)	0.73 (± 0.43)	0.736
Hemoglobin (g/dl)	14.0 (± 1.6)	13.2 (± 1.5)	**0.027**
Platelets (G/l)	232.4 (± 52.9)	234.4 (± 84.5)	0.888
CRP (mg/l)	4.3 (± 3.1)	5.3 (± 3.5)	0.056
Total cholesterol (mg/dl)	208.3 (± 43.4)	193.5 (± 63.0)	0.195
LDL (mg/dl)	122.9 (± 36.0)	105.9 (± 49.9)	0.076
HDL (mg/dl)	48.0 (± 19.5)	45.0 (± 23.0)	0.415
Triglycerides (mg/dl)	168 (± 10)	177 (± 14)	0.165
HbA1c (%)	5.7 (± 0.9)	5.8 (± 1.2)	0.593

*p* values < 0.05 are shown in bold

*HCY* homocysteine, *SDMA* symmetric dimethylarginine, *ADMA* asymmetric dimethylarginine, *BMI* body mass index, *eGFR* estimated glomerular filtration rate, *ABI* ankle–brachial index, *hs*-*CRP* high-sensitive C-reactive protein, *LDL* low-density lipoproteins, *HDL* high-density lipoproteins, *HbA1c* hemoglobin A1c

**Fig. 2:**
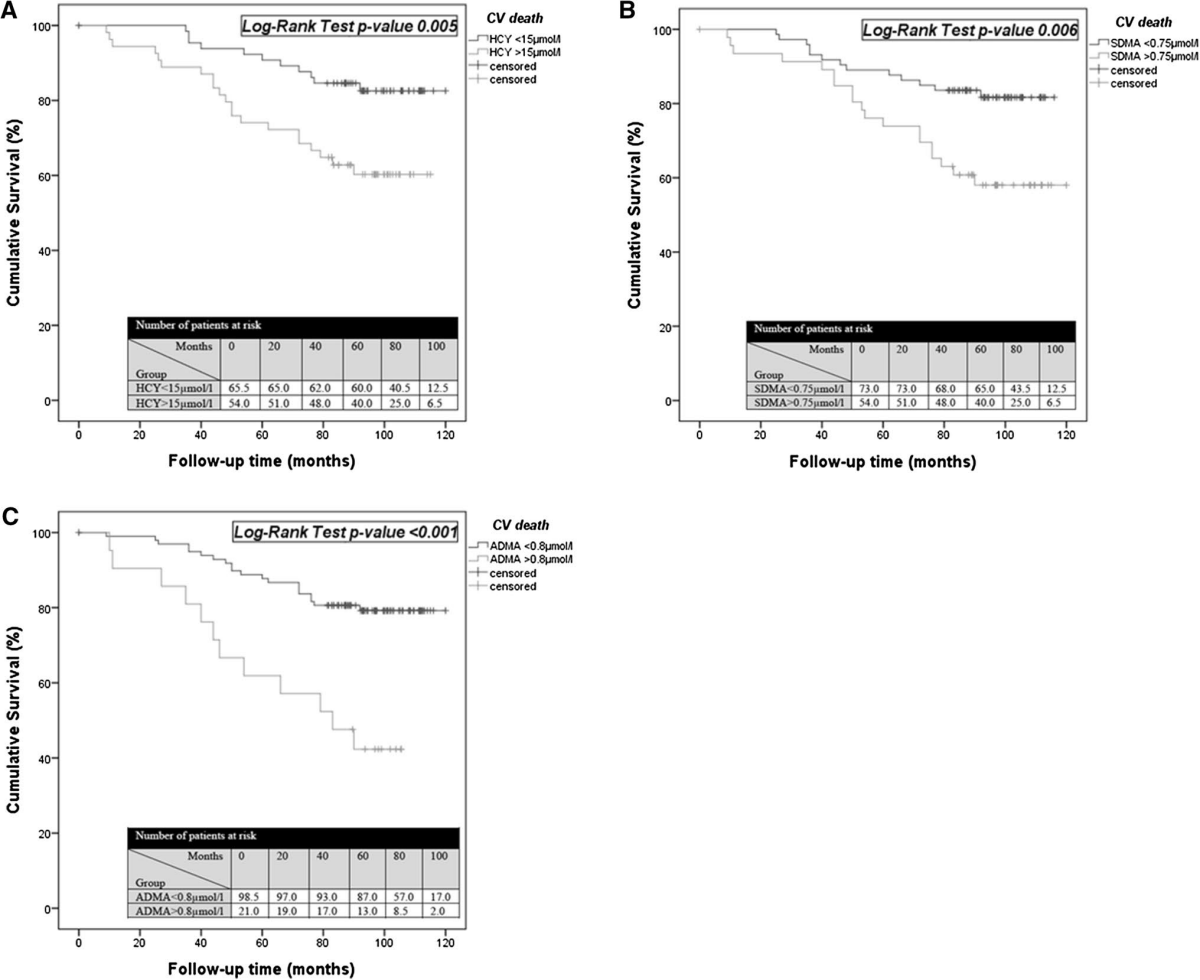
Kaplan–Meier curves for survival of CV death between HCY ≤/>15 µmol/l (**a**), SDMA ≤/> 0.75 µmol/l (**b**), and ADMA ≤/> 0.8 µmol/l (**c**)

During the observation period, in the HCY group ≤ 15 µmol/l 11/66 patients (16.3%), and in the HCY group > 15 µmol/l 21/54 patients (38.9%) died as a result of CV disease (*p* = 0.007). Furthermore, survival from CV disease was significantly shorter in the group with HCY ≤ 15 µmol/l as compared to the one with HCY > 15 µmol/l (109.5 ± 3.0 months [95% CI 103.5–115.4] vs. 88.9 ± 4.9 months [95% CI 79.4–98.4]; log-rank test *p* = 0.005). In the group with SDMA ≤ 0.75 µmol/l 13/73 patients (17.8%) and in the group with SDMA > 0.75 µmol/l 19/47 patients (40.4%) died as a consequence of CV disease (*p* = 0.010). The mean survival duration of a patient with CV disease was significantly different between the two SDMA groups, as well (SDMA ≤ 0.75 µmol/l: 104.3 ± 3.1 months [95% CI 98.2–110.3]; SDMA > 0.75 µmol/l 92.0 ± 5.4 months [95% CI 81.4–102.6]; log-rank test *p* = 0.006). Similar observations could be made in case of ADMA. In the group with ADMA ≤ 0.8 µmol/l 20/99 patients (20.2%), and in the group with ADMA > 0.8 µmol/l 12/21 patients (57.1%) died as a result of CV disease (*p* = 0.002). The mean survival duration was also significantly different in case of ADMA (ADMA ≤ 0.8 µmol/l: 106.4 ± 2.9 months [95% CI 100.8–112.0]; ADMA > 0.8 µmol/l 73.0 ± 7.4 months [95% CI 58.4–87.5]; log-rank test *p* < 0.001) (Table 4, Fig. 2).

**Table 4:** Multivariable linear regression model for CV death

Risk factor	Beta coefficient	95% CI	*P* value
Age	0.413	0.007–0.028	**0.001**
eGFR	0.223	− 0.001–0.009	0.088
SDMA	0.188	− 0.015–0.602	0.062
ADMA	0.158	− 0.081–1.235	0.085
HCY	0.012	− 0.016–0.018	0.894

*p* value < 0.05 is shown in bold

*eGFR* estimated glomerular filtration rate, *SDMA* symmetric dimethylarginine, *ADMA* asymmetric dimethylarginine, *HCY* homocysteine

**Fig. 3:**
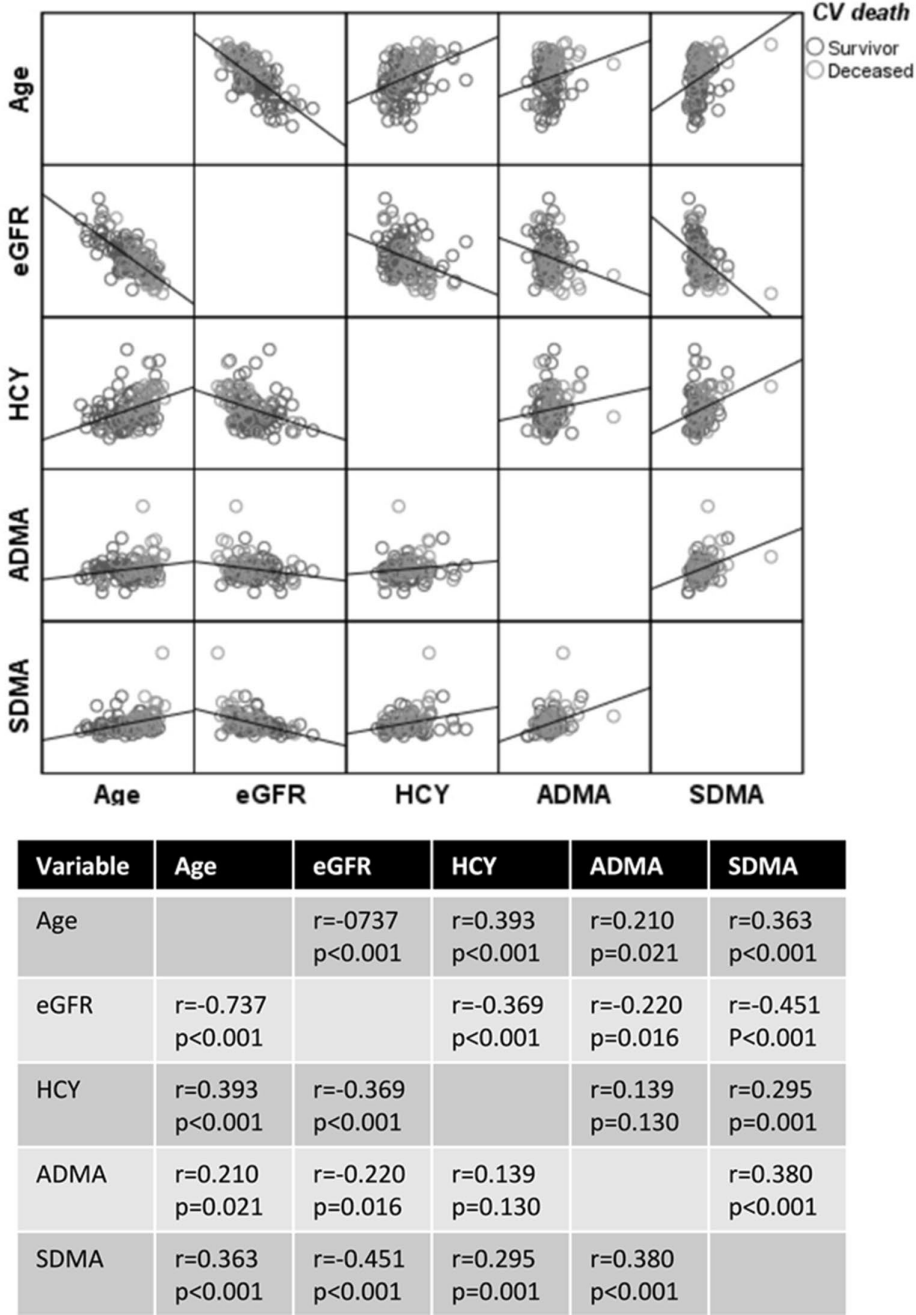
Correlation matrix between Age/eGFR/HCY/SDMA/ADMA

As a next step, the variables age, estimated glomerular filtration rate (eGFR), HCY, SDMA, and ADMA were analyzed in their metric forms regarding their associations among each other via Pearson’s correlations. Thereby, the variable age correlated significantly with HCY (*r* = 0.393; *p* < 0.001) as well as SDMA (*r* = 0.363; *p* < 0.001) and ADMA (*r* = 0.210; *p* = 0.02). A significant correlation was also observed between HCY and SDMA (*r* = 0.295; *p* = 0.001) as well as SDMA and ADMA (*r* = 0.380; *p* < 0.001). HCY and ADMA had no significant correlation (*r* = 0.139; *p* = 0.130) (Fig. 3).

**Fig. 4:**
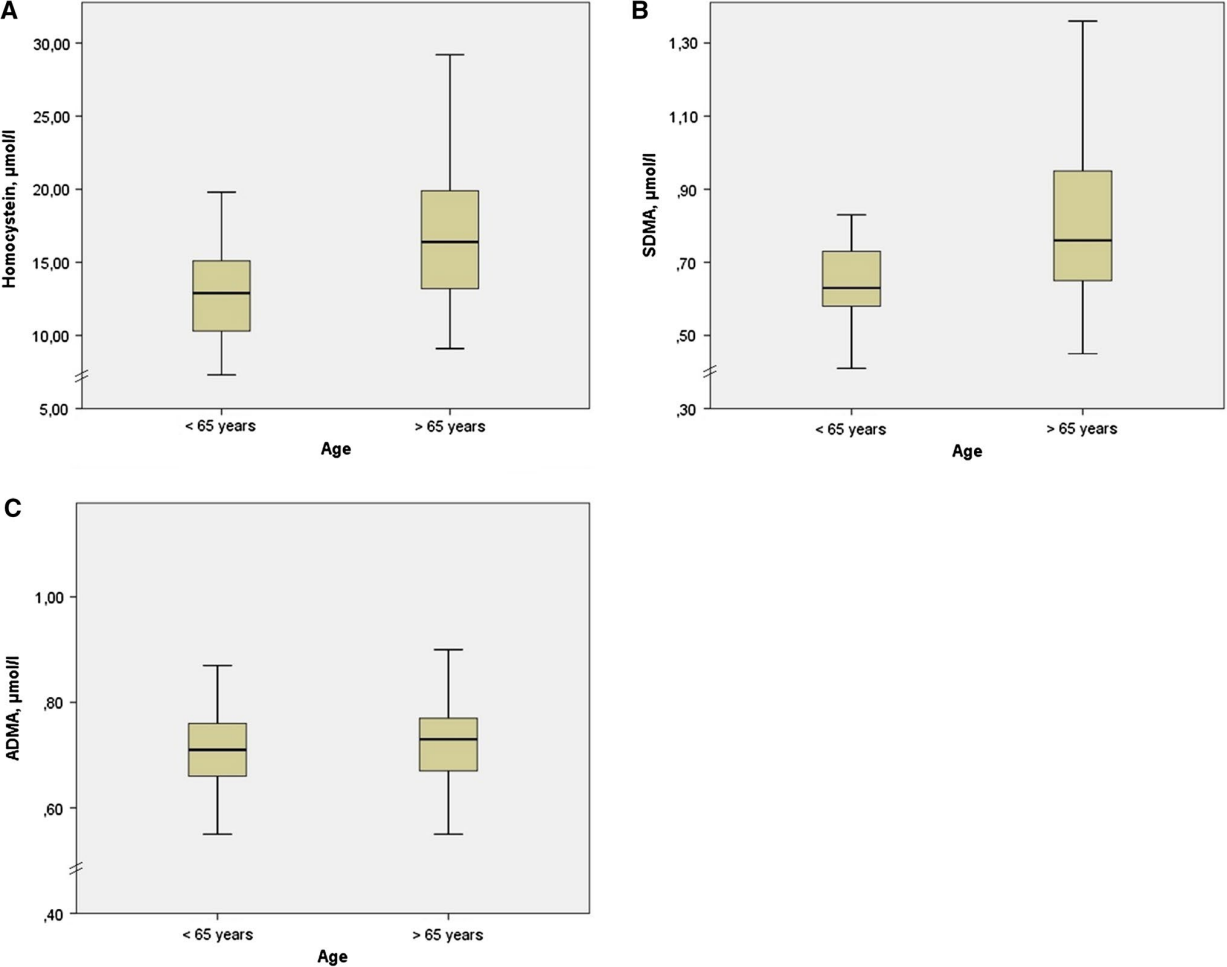
Box plots for HCY (**a**), SDMA (**b**), and ADMA (**c**) concentrations between age cutoff ≤/> 65 years

Due to this age correlation, we analyzed the age dependency of HCY, SDMA, and ADMA concentrations by splitting the cohort into two subgroups. In subjects aged below 65 years (*n* = 54, mean age 56.8 ± 6.79 years), the concentrations of HCY and SDMA were significantly lower than in those over 65 years (*n* = 66, mean age 74.44 ± 5.37 years) with median HCY 12.9 µmol/l (interquartile range 10.2–15.1) vs. 16.4 µmol/l (13.1–20.1) (*p* < 0.001) and median SDMA 0.63 µmol/l (0.58–0.73) vs. 0.76 µmol/l (0.65–0.96) (*p* = 0.01). There was no significant difference in ADMA regarding this age cutoff with median ADMA 0.71 µmol/l (0.66–0.76) vs. 0.73 µmol/l (0.67–0.77) (*p* = 0.133) (Fig. 4).

Finally, in adjusted cox proportional regression analyses including the variables age, eGFR, HCY, SDMA, and ADMA, age was independently predictive of all-cause death, when comparing the highest with the lowest tertile (HR with 95% confidence interval: 5.140 (1.191–22.187), *p* = 0.028).

## Discussion

HCY is a chemical intermediate for the metabolism of sulfurous amino acids. In case of a reaction between methionine and adenosine triphosphate, HCY is created after methylation. Methionine is supplied by the intake of food, in particular by animal products such as meat and cheese. Usually, HCY is remethylized into methionine with the help of enzymes, or metabolized into cysteine and glutathione [17]. A great variety of factors for the development of hyperhomocysteinemia have been discussed. Next to genetic enzyme defects in HCY metabolism, hyperhomocysteinemia is also in inverse correlation to the vitamin status of folic acid, vitamin B6, and vitamin B12. Chronic alcohol consumption and gastrointestinal pathologies with impaired absorption and malnutrition can lead to hyperhomocysteinemia as a result of functional deficiencies. High coffee intake was also associated with elevated HCY levels [18]. Conversely, inhibition of the proliferation of smooth muscle cells as well as a reduction of endothelial cell destruction as a result of lowering HCY levels, for instance via folic acid substitution, were reported [19, 20]. Numerous studies have determined HCY as independent cardiovascular biorisk factor. The most significant role in this context seems to be played by primarily the HCY-dependent endothelial dysfunction [21]. It is, however, unclear whether HCY directly leads to endothelial dysfunction, or if the HCY-activated dimethylarginines are responsible for it. The present study could prove a significant correlation between HCY and SDMA, but not between HCY and ADMA. On the other hand, the dimethylarginines SDMA and ADMA were significantly correlated to each other. Therefore, an HCY-dependent endothelial dysfunction via dimethylarginines appears at least partially plausible. Other data support the result of our study because dimethylarginines have already been attributed with a certain quantification of the HCY-dependent endothelial dysfunction [8, 9]. The HCY-dependent endothelial dysfunction seems to be a multifactorial process, nonetheless.

SDMA as structural isomer of ADMA is eliminated by the kidneys and was considered to be biochemically inactive. SDMA does not only seem to correlate with renal function, but also appears to have a close relationship with the expression of atherosclerotic lesions. Therefore, SDMA is also considered as an independent factor associated with the occurrence of cardiovascular end points such as stroke, myocardial infarction, and CV death [22, 23]. Furthermore, data that associated SDMA more strongly with CV events than ADMA have been published [24]. The most plausible underlying pathology not in this context seems to be the influence of SDMA on the store-operated calcium channels located on the endothelial cells after activation by HCY, as an activation of these channels by SDMA results in an increase of oxidative stress [8]. The effects of oxidative stress consequently lead to an increase in the expression of redox-sensitive genes which constitutes a deciding step of early atherogenesis [25, 26].

We could neither observe a significant association between HCY and ADMA nor a significant difference in ADMA values between older and younger patients, although older claudicant patients have significantly higher values of HCY (*p* < 0.001) and SDMA (*p* = 0.01) than younger patients. A possible explanation for these findings could potentially be found in the form of the enzyme dimethylarginine dimethylaminohydrolase which does not influence SDMA at all [27]. Dimethylarginine dimethylaminohydrolase is primarily responsible for an HCY-induced inhibition of the ADMA/eNOS/NO pathway in endothelial cells, but does not exhibit any significant changes with increasing age and in the presence of ischemia in particular [28, 29].

As outlined above, the present study significantly associated HCY, SDMA, and ADMA with the occurrence of CV death. After correction of the variables in a logistic regression model, the variable age was identified as the only significant risk factor and age correlated significantly with HCY, SDMA, and ADMA (*r* = 0.393; *r* = 0.363; *r* = 0.210, respectively). Therefore, increasing age significantly mitigated the prognostic importance of HCY as well as dimethylarginines as prognostic markers for long-term observations in our work. A strong influence of the variable age on these parameters via multivariate analyses could already be shown in multiple prior studies [10, 30]. Therefore, the present study only assumed an influence on the results which finally remain to be proven. It is possible that the prognostic effect of HCY, SDMA, and ADMA is overestimated in long-term observations especially as the strong association of these variables with CV events could also be explained by their significant age dependency. Therefore, we suggest that age-adjusted cutoff values for HCY and SDMA may estimate the risk for CV death more appropriately.

A limitation of our study is that it is one with a very selective patient cohort which investigated the mortality exactly for claudicant patients with Rutherford classification 2–3. On the other hand, our overall patient cohort was rather homogenous regarding the dietary intake and prior exercise therapy. Therefore, these parameters should affect the HCY metabolism in a less distinctive manner in our study, and the HCY metabolism seems to be more depending on the age than on other conditions. Nevertheless, further evaluation with larger cohort studies including patients with other types of LEAD is necessary to clarify the age dependency of HCY, SDMA, and ADMA including their age-adjusted cutoff values.

## Conclusions

In summary, the present study could prove a significant association between HCY and SDMA, but not between HCY and ADMA. Consequently, HCY-dependent endothelial dysfunction seems to be caused at least partially by dimethylarginines. Due to the distinct age dependency of HCY and SDMA in the present cohort, age-adjusted cutoff values for these parameters may be more appropriate as independent predictors of CV death.

## Abbreviations

ADMA: Asymmetric dimethylarginine
CV: Cardiovascular
eGFR: Estimated glomerular filtration rate
eNOS: Endothelial NO synthase
HCY: Homocysteine
LEAD: Lower extremity arterial disease
NO: Nitric oxide
ROC: Receiver operating curve
ROS: Reactive oxygen species
SDMA: Symmetric dimethylarginine
